# β-Asarone Inhibits Invasion and EMT in Human Glioma U251 Cells by Suppressing Splicing Factor HnRNP A2/B1

**DOI:** 10.3390/molecules23030671

**Published:** 2018-03-16

**Authors:** Li Li, Mingxia Wu, Chengqiang Wang, Zanyang Yu, Hongmei Wang, Hongyi Qi, Xiaoyu Xu

**Affiliations:** College of Pharmaceutical Sciences, Southwest University, Chongqing 400716, China; lilyli@email.swu.edu.cn (L.L.); zhangww_2005@126.com (M.W.); wangchengqiang432@163.com (C.W.); y1128y@email.swu.edu.cn (Z.Y.); wanglitcm@sina.com (H.W.)

**Keywords:** β-asarone, Invasion, epithelial-mesenchymal transition, hnRNP A2/B1, brain tumor

## Abstract

β-asarone, the main component in the volatile oil of *Acori tatarinowii* Rhizoma, has been found to possess antitumor activity. However, its effect and mechanisms against tumor invasion and epithelial–mesenchymal transition (EMT) are still unclear. In this study, no or less cytotoxicity was caused by β-asarone within 0–120 μM in human glioma U251 cells for 48 h. β-asarone (30 and 60 μM) inhibited the migration of U251 cells in the wound healing assay, suppressed the invasion of U251 cells in the Boyden chamber invasion assay, and inhibited the adhesion of U251 cells onto the Matrigel. Moreover, β-asarone suppressed EMT with the up-regulation of E-cadherin and the down-regulation of vimentin. HnRNP A2/B1, a well-characterized oncogenic protein, was shown at a high basal level in U251 cells and β-asarone reduced hnRNP A2/B1 expression in a concentration and time-dependent way. Importantly, hnRNP A2/B1 overexpression significantly counteracted the inhibition of β-asarone on the migration, invasion, and adhesion of U251 cells and reversed the modulation of EMT markers by β-asarone. Additionally, β-asarone decreased the MMP-9 and p-STAT3 in U251 cells, which was also reversed by hnRNP A2/B1 overexpression. Together, our results suggest that hnRNP A2/B1 may be a potential molecular target underlying the inhibitory effect of β-asarone on invasion and EMT in glioma cells.

## 1. Introduction

Brain tumors are one of the most common malignant tumors and are generally associated with a dismal prognosis and poor quality of life. In the United States in 2018, it is estimated that the new diagnoses of brain and other nervous system tumors will be 23,880 and the deaths of brain and other nervous system tumors are expected to 16,830. The death rates have increased slightly for cancers of the brain and nervous system in both men and women. According to the data of 2015, the leading cause of cancer death among men below the age of 40 is brain and other nervous system tumors, and among woman below the age of 20 [1]. Gliomas are the most malignant type of brain tumor and are characterized by rapid growth, highly invasiveness, and enhanced angiogenesis [2].

Heterogeneous nuclear ribonucleoproteins A2/B1 (hnRNP A2/B1) are two structurally homologous proteins belonging to the family of hnRNPs, which are known as a group of RNA-binding proteins regulating the splicing and transportation of mRNA and participating in growth regulation and carcinogenesis [3]. A growing body of evidence has demonstrated that hnRNP A2/B1 is a novel oncogenic protein and is overexpressed in various tumors, including breast [4], pancreatic [5], liver [6], gastric [7], and lung carcinoma cells [8]. Recently, hnRNP A2/B1 overexpression has also been described in human glioma tissue specimens and is associated with advanced glioma grades [9,10]. Moreover, emerging evidence has firmly established that hnRNP A2/B1 is strongly related to the invasion and/or epithelial–mesenchymal transition (EMT) in glioma [10], lung cancer [8,11], pancreatic cancer [12], and hepatocellular carcinoma [13]. Moreover, knockdown or direct targeting of hnRNP A2/B1 has been shown to dramatically inhibit the invasion and EMT [10,11]. Thus, hnRNP A2/B1 is a key activator of invasion and EMT, and targeting hnRNP A2/B1 may provide potential approaches for the therapeutic intervention of glioma and other relevant tumors.

**Figure 1:**
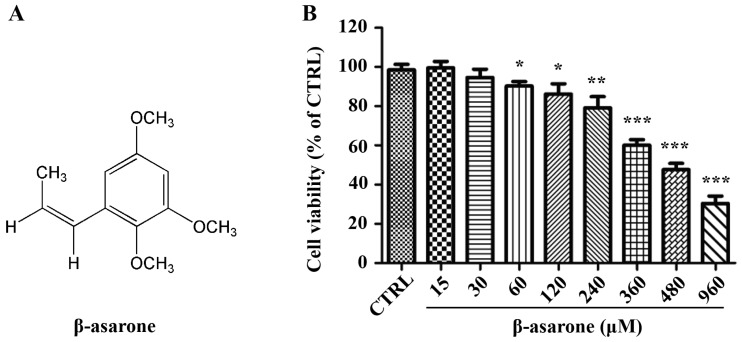
Cytotoxicity of β-asarone to U251 cells. (**A**) Chemical structure of β-asarone; (**B**) Cells were treated with β-asarone as indicated for 48 h, and cell viability was determined by sulforhodamine B assay and expressed as a percentage of that of the untreated cells. Values represent mean ± SD. * *p* < 0.05, ** *p* < 0.01 and *** *p* < 0.001 compared with vehicle.

*Acori tatarinowii* Rhizoma is the dry rhizome of *Acorus tatarinowii* Schott and has been traditionally used for central nervous system (CNS) disorders in China. Our recent investigation demonstrated that volatile oil of *Acori tatarinowii* Rhizoma (VOA) exhibited potent anti-tumor activity in glioma cells [14]. Moreover, we found that β-asarone (Figure 1A), the main component in the VOA, was shown to inhibit the growth of glioma cells [15], which was further confirmed by another group [16]. However, the inhibitory effect of β-asarone on the invasion and EMT of glioma cells remains unclear. To comprehensively identify the potential molecular targets regulated by β-asarone, we applied a proteomic approach and identified hnRNP A2/B1 as one of the key protein targets [15]. It is interesting for us to further investigate the potential role of hnRNP A2/B1 in the anti-glioma effect of β-asarone.

In the current study, we first determined the inhibitory effects of β-asarone on the migration, invasion, and adhesion of human glioblastoma U251 cells and then also evaluated the influence of β-asarone on the EMT process. Moreover, we also sought to identify the underlying role of hnRNP A2/B1 and its relevant mechanisms in these processes. 

## 2. Results

### 2.1. Cytotoxicity of β-Asarone to U251 Cells

In the current study, we first evaluated the cytotoxicity of β-asarone on U251 cells. The cells were treated with various concentrations of β-asarone (15–960 μM) for 48 h followed by SRB assay. The result demonstrated that no or less toxicity was observed in U251 cells treated by β-asarone at concentrations of 15–120 μM, while higher toxicity was shown at concentrations of 240–960 μM (Figure 1B).

### 2.2. β-Asarone Inhibits Migration, Invasion, and Adhesion of U251 Cells

**Figure 2:**
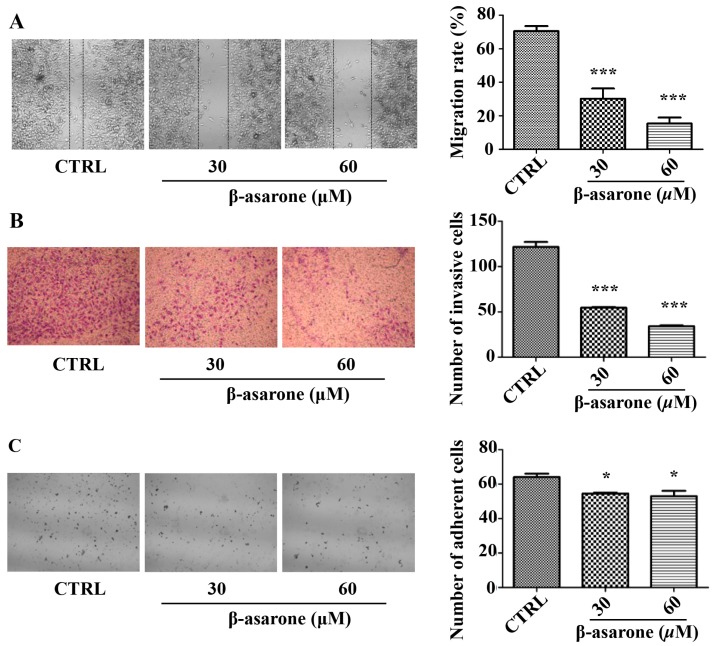
β-asarone suppressed the migration, invasion, and adhesion of U251 cells. (**A**) Wound healing assay. Cells were seeded on each side of an Ibidi culture insert overnight and then treated with β-asarone as indicated for 48 h, respectively. Cells were photographed following 0, 12, 24, 36, and 48 h of incubation. The representative images were captured at 48 h after treatment; (**B**) Boyden chamber invasion assay. Cells were pretreated with β-asarone as indicated for 24 h and then seeded in the chamber with coated Matrigel of a 24-transwell plate for another 24-h invasion assay; (**C**) Adhesion assay. Cells were preincubated with β-asarone as indicated for 24 h and seeded onto a 96-well plate coated with Matrigel for 4-h adhesion. Then, attached cells were stained and counted. Randomly chosen fields were obtained using an optical microscope (100× magnification). Values represent mean ± SD. * *p* < 0.05 and *** *p* < 0.001 compared with vehicle.

To determine the effect of β-asarone on the migration capability of U251 cells, we performed the wound healing assay with an Ibidi culture insert. Cells were exposed to β-asarone with the concentrations of 30 and 60 μM to exclude the potential interference of cytotoxicity. Wound closure was monitored and photographed at 0, 12, 24, 36, and 48 h. As representative fields shown in Figure 2A, after treatment for 48 h, β-asarone with 30 and 60 μM concentration-dependently inhibited the flattening and spread of U251 cells along the edges of the wound compared to control (*p* < 0.001). Furthermore, the invasion assay was performed in U251 cells using Matrigel-coated 24-well Boyden chambers. Figure 2B showed that β-asarone (30 and 60 μM) remarkably inhibited the invasion of U251 cells compared to control (*p* < 0.001). Finally, the influence of β-asarone on the adhesion of U251 cells was determined. As shown in Figure 2C, the adhesion of U251 cells onto the Matrigel was moderately inhibited by β-asarone with 30 and 60 μM (*p* < 0.05). These results revealed that β-asarone exhibited a significant inhibitory effect on the migration, invasion, and adhesion of U251 cells.

### 2.3. β-Asarone Prevents the EMT Process of U251 Cells

**Figure 3:**
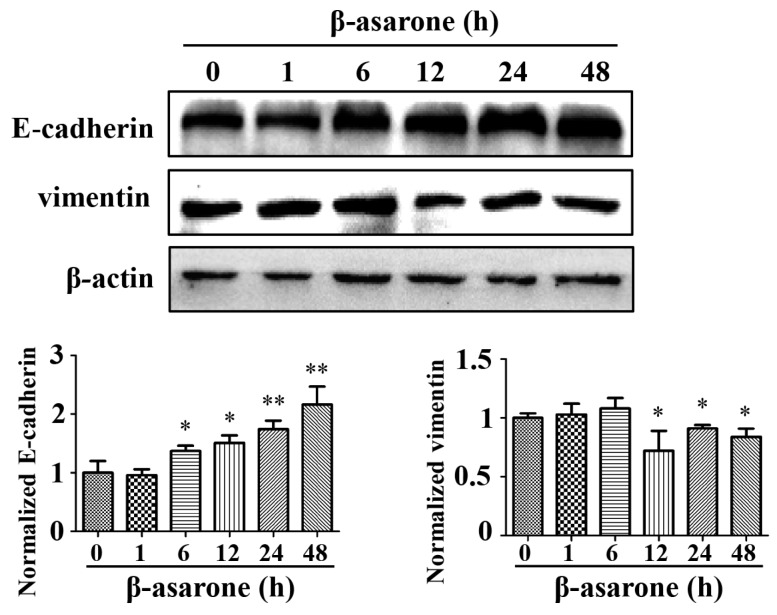
β-asarone modulated the expression of EMT markers E-cadherin and vimentin. Cells were treated with β-asarone (60 μM) for the indicated time and the expression of E-cadherin and vimentin was analyzed by Western blotting. The blots were representative of three independent experiments. * *p* < 0.05 and ** *p* < 0.01 compared with vehicle.

Since human glioma malignancy is highly related to EMT, we then investigated whether β-asarone can inhibit EMT. Western blotting was applied to determine the influence of β-asarone on the expression of specific EMT markers after U251 cells were treated by β-asarone for different times. As a result, we found that β-asarone increased the expression of E-cadherin in a time dependent manner within 48-h treatment, while it reduced vimentin at 12, 24, and 48 h (Figure 3).

### 2.4. β-Asarone Suppresses Expression of HnRNP A2/B1 in U251 Cells

**Figure 4:**
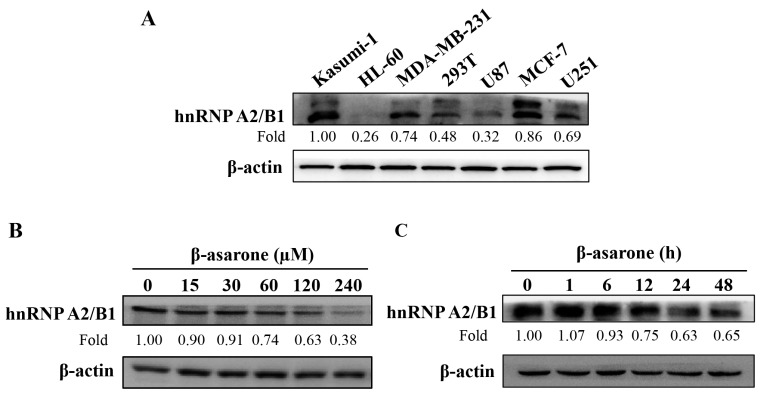
Analysis of hnRNP A2/B1 protein expression. (**A**) Detection of hnRNP A2/B1 protein expression in various cell lines. The cell lines as indicated were seeded and cultured for 24 h; (**B**,**C**) Effect of β-asarone on the expression of hnRNP A2/B1 in U251 cells. Cells were treated with β-asarone with different concentrations for 24 h or with 60 µM β-asarone for different times, as indicated. The expression of hnRNP A2/B1 was determined by Western blotting. The blots were representative of three independent experiments.

As accumulating evidence indicated that hnRNP A2/B1 played a pivotal role in increasing the metastatic propensity of tumor cells [9,10], we thereafter payed special attention to hnRNP A2/B1. First, we determined the protein expression of hnRNP A2/B1 using a specific antibody in a group of human cell lines. As shown in Figure 4A, a relatively high expression of hnRNP A2/B1 protein was detected in Kasumi-1 and MCF-7 cells and a moderate level was shown in MDA-MB-231 and U251 cells, whereas a very weak level was shown in U87 and HL-60 cells. Moreover, U251 and U87 cells also exhibited a higher level of hnRNPA2 (34 k, lower band) than its splicing variant hnRNPB1 (36 k, upper band). Then, we examined the protein expression of hnRNP A2/B1 after 24 h treatment of U251 cells by vehicle or β-asarone (30–480 μM). Figure 4B showed that basal protein expression of hnRNP A2/B1 was observed in U251 cells treated with vehicle, whereas the protein expression of hnRNP A2/B1 was remarkably reduced after β-asarone (30–240 μM) treatment for 24 h. Furthermore, β-asarone (60 μM) substantially decreased hnRNP A2/B1 protein expression as early as 12 h after treatment (Figure 4C).

### 2.5. HnRNP A2/B1 Overexpression Mitigates the Inhibitory Effect of β-Asarone on Migration, Invasion, and Adhesion of U251 Cells

**Figure 5:**
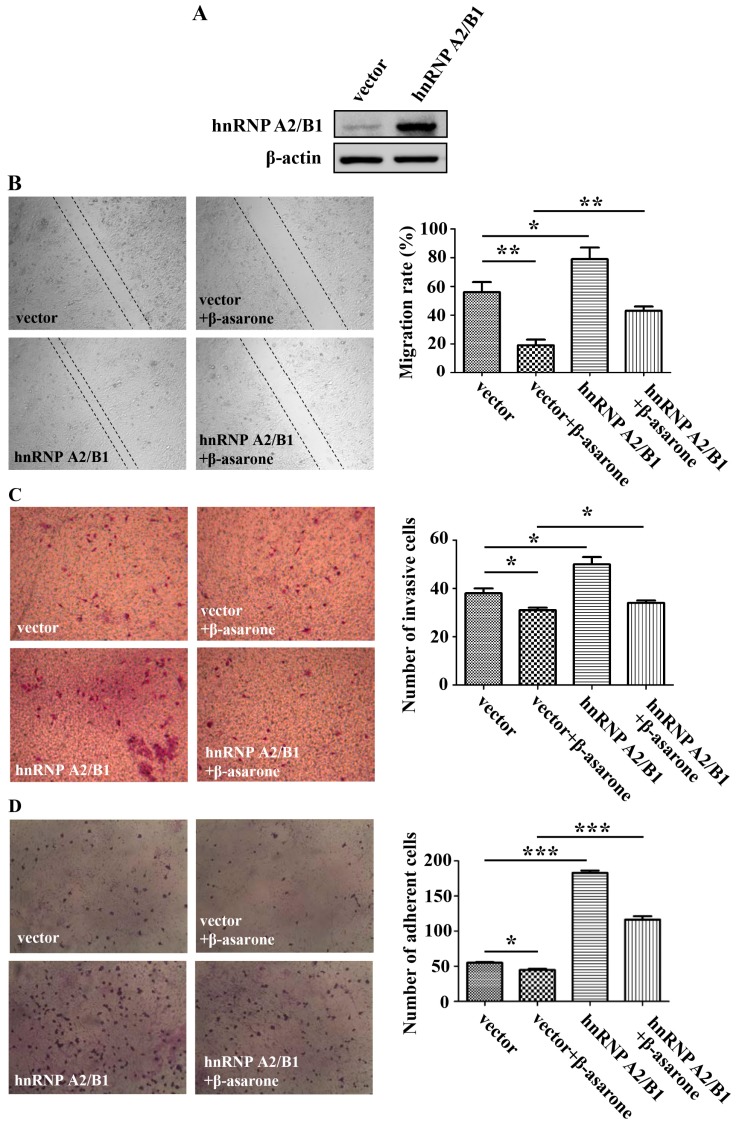
hnRNP A2/B1 overexpression reversed the inhibitory effect of β-asarone on migration, invasion, and adhesion. (**A**) The level of hnRNP A2/B1 was measured by Western blotting after U251 cells were transfected with pCMV3 or pCMV3-hnRNP A2/B1 for 48 h; (**B**) Migration assay. After being transfected with pCMV3 or pCMV3-hnRNP A2/B1 for 24 h, cells were seeded on each side of an Ibidi culture insert overnight and then treated with β-asarone (60 μM) or vehicle for 48 h; (**C**) Boyden chamber invasion assay. After being transfected with pCMV3 or pCMV3-hnRNP A2/B1 for 24 h, U251 cells were pretreated with β-asarone (60 μM) or vehicle for 24 h and then seeded in the chamber with coated Matrigel of a 24-transwell plate for another 24-h invasion assay; (**D**) Adhesion assay. After being transfected with pCMV3 or pCMV3-hnRNP A2/B1 for 48 h, U251 cells were preincubated with β-asarone (60 μM) or vehicle for 24 h and seeded onto a 96-well plate coated with Matrigel for 4-h adhesion. Then, attached cells were stained and counted. Randomly chosen fields were obtained using an optical microscope (100× magnification). Values represent mean ± SD. * *p* < 0.05, ** *p* < 0.01, and *** *p* < 0.001.

To verify the potential role of hnRNP A2/B1 in the inhibitory effect of β-asarone on migration, invasion, and adhesion, we first transfected U251 cells with vector or pCMV3-hnRNP A2/B1. As shown in Figure 5A, remarkable overexpression of hnRNP A2/B1 protein was observed in U251 cells with pCMV3-hnRNP A2/B1 transfection compared to that with vector transfection. Then, the migration, invasion, and adhesion assays were performed after U251 cells were first transfected with vector or pCMV3-hnRNP A2/B1 and then treated with β-asarone or vehicle. As shown in Figure 5B–D, overexpression of hnRNP A2/B1 significantly promoted the migration (*p* < 0.05), invasion (*p* < 0.05), and adhesion (*p* < 0.001) of U251 cells compared to that of vector. Notably, the migration assay with an Ibidi culture insert (Figure 5B) showed that the inhibition of migration by β-asarone in U251 cells was obviously reversed by overexpression of hnRNP A2/B1 (*p* < 0.01). Moreover, it seems that the inhibitory effect of β-asarone on the invasion and adhesion was also mitigated to an extent by overexpression of hnRNP A2/B1 (Figure 5C,D).

### 2.6. HnRNP A2/B1 Overexpression Alleviated the Preventive Effect of β-Asarone on the EMT Process of U251 Cells

**Figure 6:**
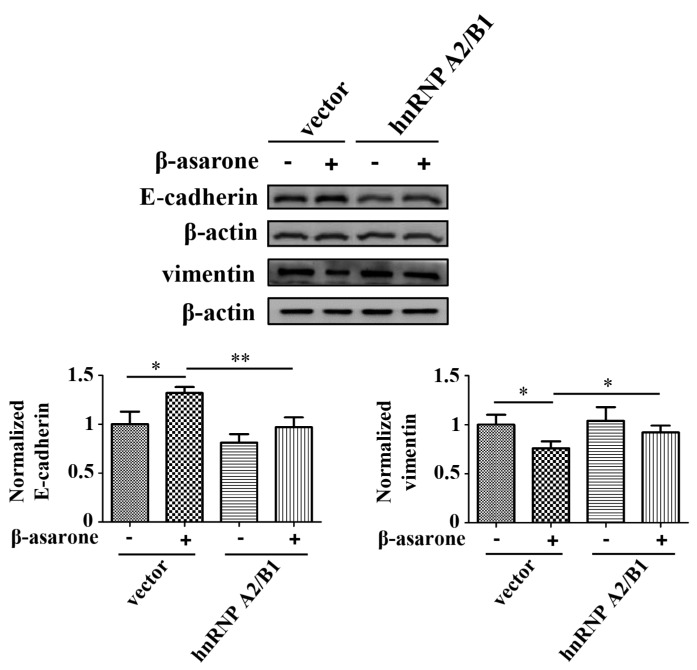
hnRNP A2/B1 overexpression counteracted the effect of β-asarone on EMT markers in U251 cells. Cells were transfected with pCMV3 or pCMV3-hnRNP A2/B1 for 48 h and then treated with β-asarone (60 μM) or vehicle for 24 h. The expression of E-cadherin and vimentin was analyzed by Western blotting. The blots were representative of three independent experiments. * *p* < 0.05 and ** *p* < 0.01 compared with vehicle.

To determine the influence of hnRNP A2/B1 on the inhibition of EMT process by β-asarone, we examined the expression change of specific EMT markers. As shown in Figure 6, the induction of E-cadherin protein by β-asarone was obviously blocked with the overexpression of hnRNP A2/B1 (*p* < 0.01), while the suppression of vimentin protein by β-asarone was significantly alleviated with the overexpression of hnRNP A2/B1 (*p* < 0.05).

### 2.7. MMP-9 and p-STAT3 Expression is Related to Inhibitory Effect of β-Asarone on HnRNP A2/B1

**Figure 7:**
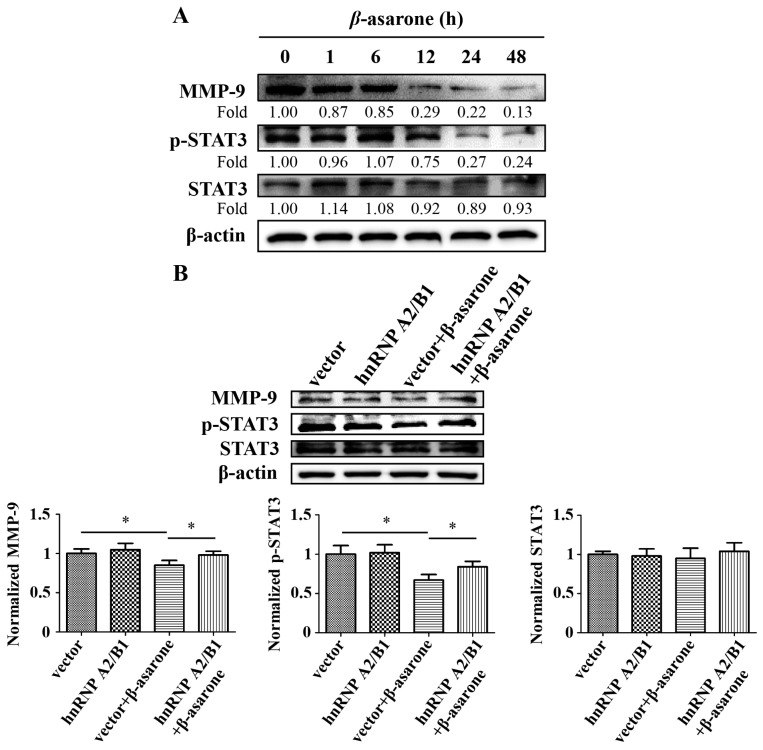
**Figure** **7.** β-asarone inhibited the expression of the downstream proteins MMP-9 and p-STAT3 of hnRNP A2/B1 in U251 cells. (**A**) Cells were treated with β-asarone (60 μM) for the indicated time; (**B**) Cells were transfected with pCMV3 or pCMV3-hnRNP A2/B1 for 48 h and then treated with β-asarone (60 μM) or vehicle for 24 h. The protein expression was analyzed by Western blotting. The blots were a representative of three independent experiments. * *p* < 0.05 compared with vehicle.

To explore the molecular events downstream of hnRNP A2/B1, we first determined the expression of matrix metalloproteinases (MMPs) and STAT3, which are highly associated with the invasion and EMT of tumor cells and are modulated by hnRNP A2/B1 [10,17,18,19], after U251 cells were treated with β-asarone (60 µM) for different times. Our Western blotting results (Figure 7A) showed that β-asarone significantly reduced the MMP-9 protein expression in U251 cells at timepoints 12, 24 and 48 h. Moreover, β-asarone also significantly reduced the expression of p-STAT3 in U251 cells at timepoints 24 and 48 h. Furthermore, we determined the role of hnRNP A2/B1 in the down-regulation of MMP-9 and p-STAT3 by β-asarone. As shown in Figure 7B, our results demonstrated that the decrease of MMP-9 and p-STAT3 by β-asarone was obviously mitigated in U251 cells transfected with pCMV3-hnRNP A2/B1 compared with those transfected with vector only (*p* < 0.05).

## 3. Discussion

Glioma is regarded as one of the deadliest tumors and is refractory to present therapeutic strategies [20]. Although surgery is still the preferred approach for glioma in clinic practice, the residual tumor can invade nearby normal brain tissue, subsequently leading to high rates of recurrence. Thus, inhibition of invasion might be a complementary therapeutic method for gliomas [21,22]. β-asarone, the major component in the VOA, has been shown to not only directly enter blood-brain barrier (BBB), but also improve the permeability of BBB and inhibit the function of P-glycoprotein [23,24,25]. Recent results from our group [15] and others [16] revealed that β-asarone effectively inhibited the growth of glioma cells. However, its effect on the invasion of glioma cells is still unknown. In this study, we first discovered that β-asarone remarkably blocked the migration of U251 cells. Furthermore, we found that β-asarone exhibited a significant inhibitory effect on the Boyden chamber invasion and the adhesion of highly metastatic U251 cells, suggesting that β-asarone has the potential for inhibiting invasion of glioma cells. Epithelial to mesenchymal transition (EMT), a process whereby epithelial cells transform into mesenchymal cells, is frequently observed in subsets of carcinoma cells undergoing phenotypic conversion for metastasis [26]. The typical characteristics of EMT are the loss of epithelial cell junction proteins, such as E-cadherin, and the gain of mesenchymal markers, such as vimentin [26,27]. It is known that EMT enhances the invasiveness and migratory potential of tumor cells and promotes their malignant transformation, especially in malignant gliomas [27]. Thus, we further determined the influence of β-asarone on EMT. The up-regulation of E-cadherin level and the down-regulation of vimentin were observed in U251 cells after β-asarone treatment, suggesting β-asarone may block the process of EMT in glioma cells.

Recently, a two-dimensional gel electrophoresis (2-DE)-based proteomic strategy was applied in our study to systematically identify the potential proteins regulated by β-asarone in U251 cells [15]. Among the successfully identified proteins, hnRNPA2/B1 drew our special attention. In this study, we first examined the expression level of hnRNPA2/B1 in a group of human cell lines. As a result, hnRNPA2/B1 was detected at various levels, whereas U251, Kasumi-1, and MCF-7 cells showed a higher level of hnRNPA2/B1 than their counterparts U87, HL-60, and MDA-MB-231 cells. Although hnRNPA2/B1 has been shown as an oncogenic protein with relatively high expression level in both U251 [10] and U87 [9], two representative glioma cells, each cell line was used in a separated report without simultaneous comparison. Our results for the first time indicated that a higher hnRNPA2/B1 level was observed in U251 cells than that in U87 cells. Moreover, there was a higher level of hnRNPA2 than its splicing variant hnRNPB1 in U251 and U87 cells. It has been described that hnRNP A2 is oncogenic, but its splicing variant hnRNP B1 was inactive as an oncogene and could not induce transformation although it is only 36 bases longer [28,29]. Notably, U87 exhibited higher metastatic potential to lungs or other organs than U251 when grown orthotopically [30]. It seems that the level of hnRNPA2/B1 in these two cell lines is not in accordance with their aggressiveness. The reason may be that the underlying mechanisms for the aggressiveness of glioma cells are complicated and other proteins may also play a potential role in the aggressiveness of U87 and U251 cells. In our previous study, we have compared the expression of eEF1A1, a well-characterized actin-binding protein associated with the invasiveness of cancer cells, in this panel of cell lines. The results showed that the level of eEF1A1 in U87 cells is higher than that in U251 cells [10]. Previous study has demonstrated that CD44-positive Kasumi-1 exhibited higher adhesion ability than CD44-negative HL-60 cells [31]. Notably, CD44 has been described as co-expressed with hnRNP B1 in non-small cell lung cancers [32]. A recent study demonstrated that hnRNPA2/B1 expression in MCF-7 is higher than MDA-MB-231 cells, both of which are higher than that in the non-tumorigenic epithelial cell line MCF-10A [17]. It is worth noting that a moderate level of hnRNPA2/B1 has also been observed in the normal cell line HEK 293T, which is supported by previous reports showing the existence of hnRNPA2/B1 in non-malignant normal cells [33,34]. Then, we further characterized the influence of β-asarone on hnRNPA2/B1 protein level. Expectedly, β-asarone obviously inhibited the expression of hnRNPA2/B1 in a concentration and time-dependent way.

Accumulating evidence has indicated that hnRNPA2/B1 plays a pivotal role in increasing the metastatic propensity of tumor cells [10,35]. Notably, hnRNPA2/B1 has also been reported as an inducer of EMT in liver [13], pancreatic [36], and lung cancer cell lines [30]. Thus, the correlation between the β-asarone-mediated down-regulation of hnRNPA2/B1 and the inhibition of invasion and EMT in glioma cells was further explored in our subsequent investigation. Firstly, we overexpressed hnRNPA2/B1 in U251 cells with pCMV3-hnRNPA2/B1 transfection. Then, the influence of hnRNPA2/B1 overexpression and/or β-asarone intervention on the migration, invasion, and adhesion abilities of U251 cells was examined. It was found that overexpression of hnRNPA2/B1 enhanced the migration, invasion, and adhesion abilities of U251 cells, suggesting the key role of hnRNPA2/B1 in the increased metastatic propensity, which is consistent with previous investigations [9,10]. Importantly, overexpression of hnRNPA2/B1 reversed the blockage of β-asarone on the migration, invasion, and adhesion of U251 cells, suggesting that hnRNPA2/B1 may be a critical target protein responsible for the inhibitory effect of β-asarone on migration, invasion, and adhesion. Similarly, our results also showed that overexpression of hnRNPA2/B1 reversed the modulation of β-asarone on the EMT markers E-cadherin and vimentin, indicating the key role of hnRNPA2/B1 in the suppression of EMT by β-asarone. In our study, we found that the inhibitory effect of β-asarone on the invasion (Figure 5C) and the expression of MMP-9 and p-STAT3 (Figure 7B) was alleviated in U251 cells transfected with the vector only. The possible reason may be that the transfection process with Lipofectamine 2000 as the transfection reagent influenced the status of U251 cells. Finally, we explored the potential molecular events associated with the down-regulation of hnRNP A2/B1 by β-asarone. Matrix metalloproteinases (MMPs), as proteolytic enzymes, play a critical role in the breakdown of the brain extracellular matrix [18]. STAT3 is an oncogenic transcription factor and its aberrant activation has been involved in not only oncogenesis, but also tumor metastasis and EMT [19]. Both MMPs and STAT3 were found to be constitutively expressed in gliomas and highly correlated with their aggressiveness [37,38]. Importantly, it has been described that both MMPs and STAT3 are involved in the hnRNP A2/B1-related cancer metastasis [10,17]. Our results demonstrated that β-asarone reduced the expression of MMP-9 and p-STAT3 in U251 cells, while overexpression of hnRNP A2/B1 mitigated this effect of β-asarone.

## 4. Materials and Methods

### 4.1. Chemicals and Reagents

β-Asarone was purchased from Dalian Meilun Biological Technology Co., Ltd. (Dalian, China) with purity more than 98% and dissolved in dimethyl sulfoxide (DMSO) and stored at −20 °C before use. The antibodies against hnRNP A2/B1, p-STAT3, and total-STAT3 were obtained from Cell Signaling Technology (Boston, MA, USA). The antibodies against E-cadherin, vimentin, MMP-9, and β-actin were obtained from Wanlei Biotechnology (Shenyang, China). Other chemicals were obtained from Sigma-Aldrich, unless indicated otherwise.

### 4.2. Cell Culture

Human glioma U251 and U87 cells, human breast cancer MDA-MB-231 and MCF-7 cells, and human embryonic kidneys (HEK) 293T cells were obtained from the American Type Cell Culture Collection (Manassas, VA, USA) and maintained in Dulbecco’s modified Eagle’s medium (DMEM) supplemented with 10% fetal bovine serum (FBS) (Invitrogen, Carlsbad, CA, USA), 1% penicillin/streptomycin (Invitrogen, Carlsbad, CA, USA) at 37 °C in a 5% CO_2_ and 95% air atmosphere. Acute myeloid leukemia cell lines HL-60 and Kasumi-1 were purchased from Cell Bank of Chinese Academic of Science (Shanghai, China) and grown in RPMI-1640 medium (Gibco Life Technologies, Carlsbad, CA, USA) supplemented with 10% fetal bovine serum (FBS) (Invitrogen, Carlsbad, CA, USA) and 1% penicillin/streptomycin (Invitrogen, Carlsbad, CA, USA) in a 5% CO_2_ humidified incubator at 37 °C.

### 4.3. Cell Viability Assay

Sulforhodamine B (SRB) assay was used to determine cell viability [14]. After drug treatment, U251 cells were fixed with 50% (*w*/*v*) trichloroacetic acid and incubated at 4 °C for 1 h. Then, the cells were stained with 100 μL of SRB solution for 15 min and the unbound dye was removed by washing with 1% acetic acid. The bound dye was dissolved by 150 μL of 10 mM Tris base (pH 10.5). The OD value was detected at 515 nm with a microplate reader (Biotek, Winooski, VT, USA). Cell viability was presented as a percentage of that of untreated cells.

### 4.4. Wound Healing Assay

The ibidi culture-insert (Munich, Germany) was used in this study to form a cell-free gap by seeding cells into each side of the culture-insert at a density of 3 × 10^5^ cells. The culture-insert was carefully removed with sterile tweezers after 24 h incubation. Cells were treated with β-asarone (0, 30 and 60 μM) for 48 h. Then, cells were photographed using BDS200 inverted biological microscope (Optec, Chongqing, China) at the insert removal (0 h) and following 12, 24, 36, and 48 h of drug treatment.

### 4.5. Boyden Chamber Invasion Assay

A polycarbonate filter with 8 μm pore size (Corning, Corning, NY, USA) was coated with Matrigel (BD, Franklin Lakes, NJ, USA). U251 cells were pretreated with β-asarone (0, 30, and 60 μM) for 24 h. Then, cells were seeded in the upper chamber with a density of 2 × 10^5^ cells in 200 μL of serum-free medium and 500 μL of medium containing 20% FBS was added to the lower chamber. After 24 h, the filter was fixed with 100% methanol for 20 min and stained with 0.05% crystal violet for 10 min. Then, cells in randomly chosen fields were photographed using fluorescence microscopy (OLYMPUS IX71), and invading cells were quantified by manual counting. Inhibition percentage of invading cells was quantified with untreated cells representing 100%.

### 4.6. Adhesion Assay

U251 cells were pre-incubated with β-asarone (0, 30, and 60 μM) for 24 h and seeded with a density of 1.5 × 10^5^ cells/mL onto a 96-well plate coated with Matrigel for 4 h. Attached cells were fixed in 100% methanol for 30 min and stained with 0.05% crystal violet solution for 10 min. Randomly chosen fields were obtained using fluorescence microscopy (OLYMPUS IX71). Then, attached cells were quantified by manual counting. Inhibition percentage of adhesion cells was quantified with untreated cells representing 100%.

### 4.7. Western Blotting Analysis

The cells after drug treatment were lysed with ice-cold RIPA buffer (Cell Signaling Technologies, USA) supplemented with 1% (*v*/*v*) protein inhibitor cocktail and 1 mM phenylmethylsulfonyl fluoride (PMSF). Then, proteins were separated by electrophoresis in 101–2.5% SDS-polyacrylamide gel, and subsequently transferred to polyvinylidene difluoride (PVDF) membrane. After blocked with 5% nonfat milk for 1 h at room temperature, the membranes were incubated with respective primary antibodies at 4 °C overnight. Following this, the membranes were washed, and incubated with horseradish peroxidase (HRP)-conjugated secondary antibodies at room temperature for 1 h. The blots were visualized by enhanced chemiluminescence (ECL) detection reagents (GE Healthcare, Bio-Sciences AB, Uppsala, Sweden). The blots were acquired and quantified by a gel imaging system (Tanon, Shanghai, China). The concentration of the loaded cellular proteins was normalized against the internal control β-actin, and then the value was expressed as each normalized data relative to control.

### 4.8. Plasmids and Transient Transfection

The pCMV3-hnRNPA2/B1 and pCMV3 vectors were purchased from Sino Biological Inc. (Beijing, China). Cells (50% confluence) were transfected with 2.5 μg of DNA using the transfection reagent *Lipofectamine* 2000 (Invitrogen, Carlsbad, CA, USA) following the protocol provided by the manufacturer. Transfected cells were first cultured in antibiotic-free medium for 6 h and then in fresh medium for 48 h, followed by further drug treatments.

### 4.9. Statistical Analysis

All data were presented as mean ± SD for three independent experiments. Statistical analysis was performed by two-tail Student’s *t*-test. A *p*-value of less than 0.05 was considered to be statistically significant.

## 5. Conclusions

In conclusion, β-asarone blocked the migration, invasion, and adhesion and suppressed the EMT process of glioma U251 cells. The down-regulation of hnRNP A2/B1 was found to be critical for the inhibition of invasion and EMT effects of β-asarone. MMP-9 and STAT3 were proved to be the downstream events in the β-asarone-mediated hnRNP A2/B1 inhibition. These results suggest that β-asarone inhibited the invasion and EMT of glioma cells through the modulation of the hnRNP A2/B1-related signaling pathway.
